# Recombinant BCG Expressing LTAK63 Adjuvant induces Superior Protection against *Mycobacterium tuberculosis*

**DOI:** 10.1038/s41598-017-02003-9

**Published:** 2017-05-18

**Authors:** Ivan P. Nascimento, Dunia Rodriguez, Carina C. Santos, Eduardo P. Amaral, Henrique K. Rofatto, Ana P. Junqueira-Kipnis, Eduardo D. C. Gonçalves, Maria R. D’Império-Lima, Mario H. Hirata, Celio L. Silva, Nathalie Winter, Brigitte Gicquel, Kingston H. G. Mills, Mariagrazia Pizza, Rino Rappuoli, Luciana C. C. Leite

**Affiliations:** 1 Laboratório Especial de Desenvolvimento de Vacinas, Instituto Butantan, São Paulo, SP Brazil; 2 Departamento de Imunologia, Instituto de Ciências Biomédicas, Universidade de São Paulo (USP), São Paulo, SP Brazil; 3 Laboratório de Parasitologia, Instituto Butantan, São Paulo, SP Brazil; 4 Programa de Pós-Graduação Interunidades em Biotecnologia, USP, SP Brazil; 5 Instituto de Patologia Tropical e Saúde Pública, Universidade Federal de Goiás, Goiânia, GO Brazil; 6 Easytech Soluções em Biotecnologia Ltda, Ribeirao Preto, SP Brazil; 7 Departamento de Análises Clínicas e Toxicológicas, Faculdade de Ciências Farmacêuticas, USP, SP Brazil; 8 Farmacore Biotecnologia Ltda, Ribeirão Preto, SP Brazil; 9 INRA, Université de Tours, UMR 1282, Infectiologie et Santé Publique SP, Nouzilly, France; 10 Unité de Génétique Mycobactérienne, Institut Pasteur, Paris, France; 11 Immune Regulation Research Group, School of Biochemistry and Immunology, Trinity Biomedical Sciences Institute, Trinity College Dublin, Dublin, Ireland; 12 GlaxoSmithKline, Siena, Italy

## Abstract

In order to develop an improved BCG vaccine against tuberculosis we have taken advantage of the adjuvant properties of a non-toxic derivative of *Escherichia coli* heat labile enterotoxin (LT), LTAK63. We have constructed rBCG strains expressing LTAK63 at different expression levels. Mice immunized with BCG expressing low levels of LTAK63 (rBCG-LTAK63_lo_) showed higher Th1 cytokines and IL-17 in the lungs, and when challenged intratracheally with *Mycobacterium tuberculosis* displayed a 2.0–3.0 log reduction in CFU as compared to wild type BCG. Histopathological analysis of lung tissues from protected mice revealed a reduced inflammatory response. Immunization with rBCG-LTAK63_lo_ also protected against a 100-fold higher challenge dose. Mice immunized with rBCG-LTAK63_lo_ produced an increase in TGF-β as compared with BCG after challenge, with a corresponding reduction in Th1 and Th17 cytokines, as determined by Real Time RT-PCR. Furthermore, rBCG-LTAK63_lo_ also displays protection against challenge with a highly virulent Beijing isolate. Our findings suggest that BCG with low-level expression of the LTAK63 adjuvant induces a stronger immune response in the lungs conferring higher levels of protection, and a novel mechanism subsequently triggers a regulatory immune response, which then limits the pathology. The rBCG-LTAK63_lo_ strain can be the basis of an improved vaccine against tuberculosis.

## Introduction

*Mycobacterium tuberculosis* (Mtb) the causative agent of tuberculosis remains a major worldwide health problem, responsible for over 10.4 million cases and 1.8 million deaths annually^1^. The difficulty of timely diagnosis and the requirement of many months of treatment, leads to interruptions in treatment and generates antibiotic resistance. The increasing frequency of multidrug-resistant (MDR) isolates of tuberculosis and others^2, 3^, has resulted in infections extremely difficult to treat and has been a major concern for health authorities worldwide^2^.

The current vaccine against tuberculosis, *Mycobacterium bovis* Bacillus Calmette-Guérin (BCG), is the only vaccine available. It is a safe and low cost vaccine administered to more than 4 billion individuals since it’s licensing in 1921. It can protect children efficiently against early manifestations of Mtb. However, the protective memory response induced by BCG immunization wanes in 10–20 years and it induces limited protection against adult pulmonary Mtb^3^. Hence, immunization strategies to either replace or supplement BCG are urgently needed.

Several challenges remain in Mtb vaccine development, such as the lack of immune markers and correlates of protection or the definition of mechanisms of protective immunity against Mtb. Nonetheless, some aspects of the immune response that can control the infection have been identified, such as the essential role for CD4^+^ Th1 and CD8^+^ T cells that produce IFN-γ and TNF-α and more recently, a protective role for IL-17. There is also evidence that multifunctional T cells that produce IFN-γ, TNF-α and/or IL-2 simultaneously may correlate with protection^4–6^.

Over the last decades, there have been substantial efforts towards the development of new tuberculosis vaccines, some of which have reached clinical trials^3^. Current vaccine approaches have focused on a variety of strategies, such as: (1) recombinant proteins that include dominant T cell antigens from Mtb or BCG; (2) viral vectors such as MVA, adenovirus or even DNA vaccines expressing the same T cell antigens; (3) recombinant BCG (rBCG) overexpressing T cell antigens (including phagosome-escape mutants); or (4) vaccines based on rationally attenuated Mtb or other mycobacterial species, such as *M*. *vaccae* and *M*. *smegmatis* ^3, 7–10^. There are also prime-boost strategies being tested with BCG or rBCG prime and boost with recombinant proteins, viral vectors and DNA vaccines. Most of these strategies attempt to induce the class I MHC pathway of antigen presentation by cross-priming and increase CD4^+^ Th1 and CD8^+^ T cell response against the mycobacteria^11^. More than 100 Mtb vaccine candidates have been tested in different animal models, including non-human primates, with some promising candidates currently in clinical trials^12, 13^. However, efficacy in humans has not yet been demonstrated.

We have investigated an alternative strategy for improvement of the BCG vaccine. It is well known that bacterial toxins and toxin derivatives have adjuvant properties^14, 15^. We have previously generated rBCG strains expressing tetanus toxin fragment C (FC), the mutated diphtheria toxin derivative, CRM_197_, and the genetically detoxified subunit S1 of pertussis toxin – S1PT-9K/129G, demonstrating that BCG expressing FC and CRM_197_ modulates the immune response towards Th2, whereas BCG expressing S1PT promoted a shift towards Th1 responses in several animal models^16–21^. Although it is still not clear what kind of immune responses are necessary for protection against Mtb infections, it is generally accepted that induction of potent Th1 responses will be important. Therefore, we have investigated the expression of a potent Th1-driving toxin derivative – a genetically detoxified mutant of *E*. *coli* heat labile enterotoxin, LTK63^15^, in BCG, to develop a candidate vaccine against Mtb.

LT is a very potent toxin that promotes antibody and broad T cell responses, similar to cholera toxin. When used as vaccine adjuvant, LT has been shown to enhance antigen presentation, stimulate T cell proliferation and cytokine production, and promote strong mucosal IgG and IgA antibody responses^22^. Genetic detoxification in the A subunit transforms it into a potent non-toxic mucosal adjuvant with increasing ability to induce Th1 responses^15^. Broad pre-clinical testing as mucosal adjuvant showed no toxicity in mice^23^, guinea pigs^24^ and macaques^25^, and it has an extensive clinical safety record of oral and percutaneous administration, although nasal administration has not been recommended^26^. The A subunit of LTK63 has not yet been evaluated as adjuvant and it is not expected to have the potential toxic properties ascribed to the binding B subunit when delivered intranasally.

Here we have developed new rBCG constructs expressing LTK63 derivatives at different levels using promoters with varying strengths to optimize the immune response and obtain higher protective levels against Mtb challenge. The rBCG-LTAK63 strain was shown to induce protection in several mouse models (different challenge doses and times after challenge), including challenge with a highly virulent Beijing strain.

## Results

### Construction and characterization of protection by rBGC-LTK63 and rBCG-LTAK63

**Figure 1:**
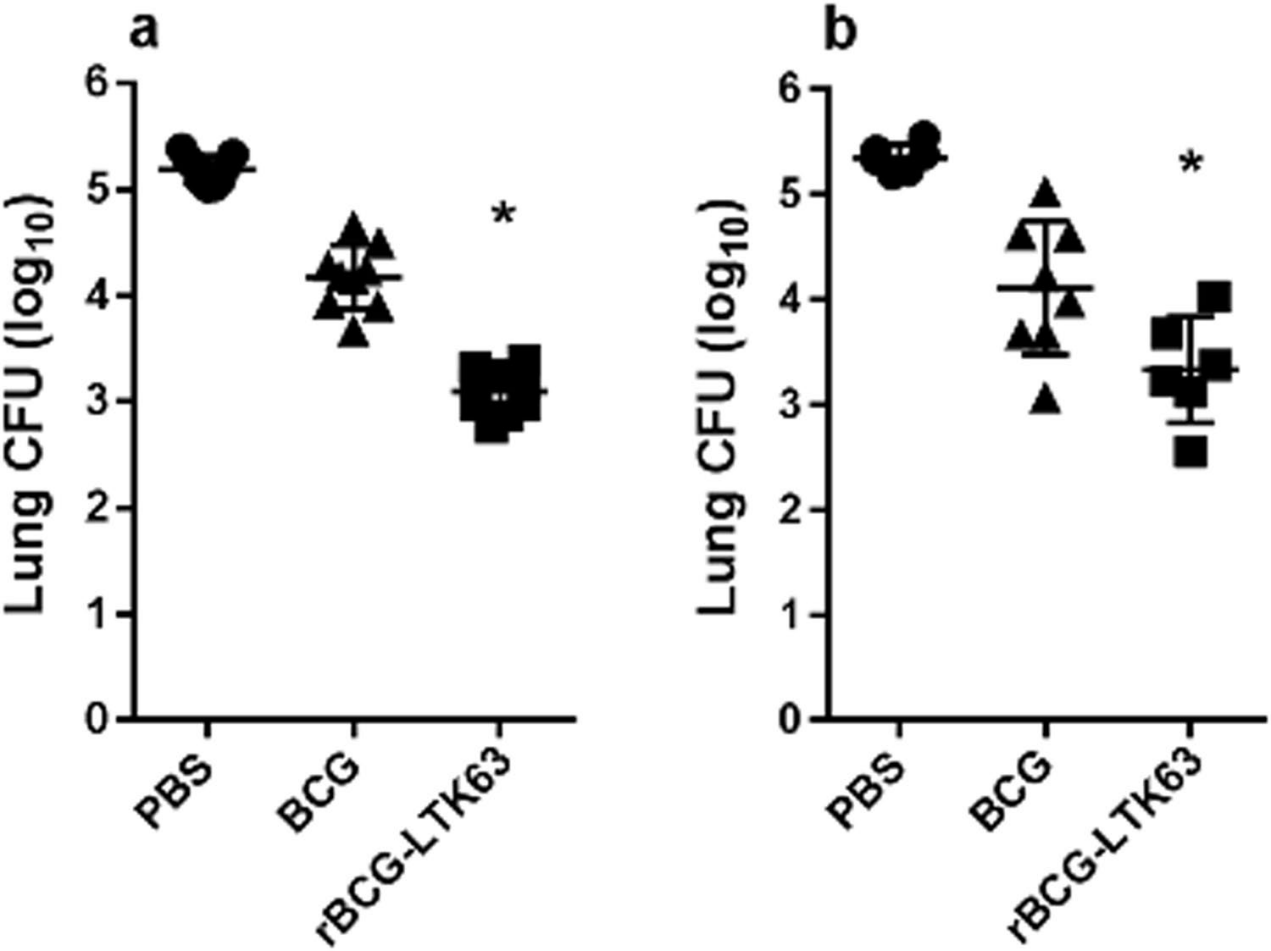
Protection of mice immunized with rBCG-LTK63 from Mtb challenge. BALB/c mice were immunized with BCG or rBCG-LTK63 (1 × 10^6^ CFU) (s.c.) and challenged intratracheally 8 weeks later with a dose of Mtb H37Rv (1 × 10^5^ CFU). Recovery of Mtb was evaluated in the lungs 30 days after challenge. (**a**) Experiment 1 (n = 10 animals) and (**b**) Experiment 2 (n = 6–8 animals). Bars are mean ± S.D. (*) Differences were considered statistically significant when *P* ≤ 0.05 as compared to the BCG group (one-way ANOVA).

Initially, the whole *ltk63* gene was placed under control of the P_blaF*_ promoter. Immunization with the rBCG-LTK63 strain induced a 2.0 log reduction in CFU in the lungs of immunized mice following a challenge with virulent *M*. *tuberculosis* H37Rv (recovered 30 days latter) when compared with control animals and a 1.0 log reduction when compared with BCG-immunized animals (Fig. 1). However, the rBCG-LTK63 strain was not stable upon passaging. We considered the hypothesis that the expression of the whole molecule could be toxic to the bacteria.

Therefore, in another construct only the *ltak63* subunit was placed under control of the P_blaF*_ promoter, resulting in a rBCG strain displaying high level of expression of LTAK63 (Supplementary Fig. 1). BALB/c mice immunized with this strain and challenged intratracheally with Mtb displayed a 1.4–2.0 log lower CFU count (recovered 30 days latter) when compared with control animals and 0.3–0.4 log reduction when compared with BCG (Supplementary Fig. 2a,b).

### Construction and protective immunity of rBCG expressing lower levels of LTAK63

**Figure 2:**
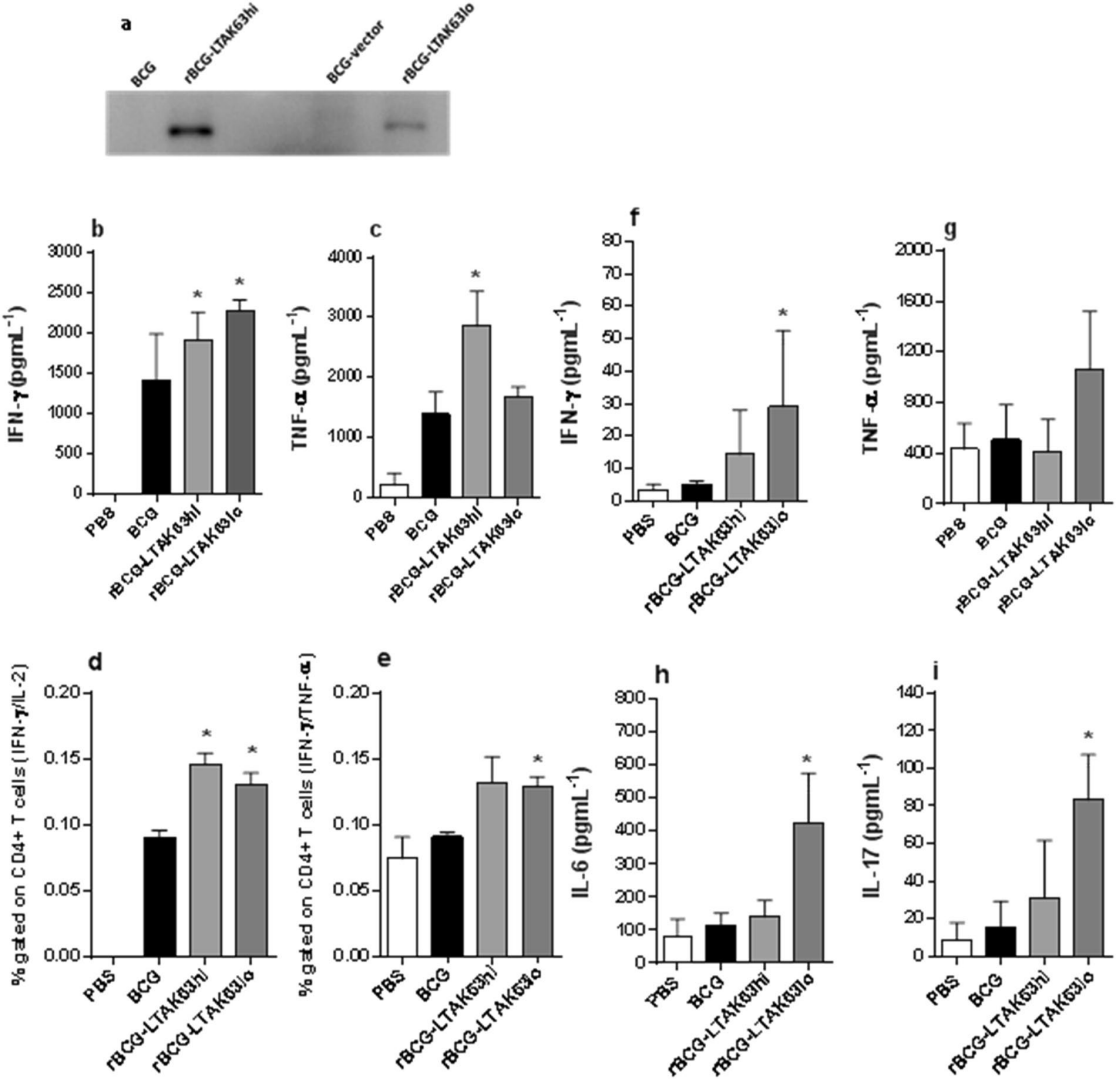
Western blot of different rBCG-LTAK63 constructs and cytokine production in the splenocytes and lungs of mice immunized with these constructs. (**a**) Western blot of total soluble protein extracts from BCG, rBCG-LTAK63_hi_, BCG-vector (BCG transformed with the empty pMIP12 expression vector) or rBCG-LTAK63_lo_ (10 μg) analyzed using a polyclonal anti-LTAK63 antibody (1:500). BALB/c mice were immunized with either BCG, rBCG-LTAK63_hi_ or rBCG-LTAK63_lo_ (1 × 10^6^ CFU). Splenocytes were isolated after 60 days and *in vitro* re-stimulated with CFP to produce (**b**) IFN-γ, and (**c**) TNF-α as determined by ELISA, or (**d**) CD4^+^ T cells doubly positive for IFN-γ/IL-2 and (**e**) CD4^+^ T cells doubly positive for IFN-γ/TNF-α. Lung cells were separated and re-stimulated with CFP to produce (**f**) IFN-γ, (**g**) TNF-α, (**h**) IL-6 and (**i**) IL-17. Results are mean ± S.D. (*) Differences were considered statistically significant when *P* ≤ 0.05 as compared to the BCG group (one-way ANOVA).

In order to evaluate if the expression levels of LTAK63 would affect the immune response and protection, the *ltak63* gene was codon optimized for expression in mycobacteria and placed under control of the P_*AN*_ promoter, considered a weaker promoter as compared to P_blaF*_. The resulting strain displayed lower expression levels of LTAK63, as compared to the original construct, generating rBCG-LTAK63_lo_ (Fig. 2a). By comparison, the construct containing the P_blaF*_ promoter was named rBCG-LTAK63_hi_ (Supplementary Fig. 1 and Supplementary Fig. 2a and b).

Immunization of BALB/c mice with rBCG-LTAK63_hi_ or rBCG-LTAK63_lo_ induced mycobacteria-specific IFN-γ-secreting spleen cells; TNF-α production was also detected in splenocytes from mice immunized with rBCG-LTAK63_hi_ (Fig. 2b,c). Furthermore, both constructs induced significantly higher production of IFN-γ/IL-2 double positive CD4^+^ T cells and rBCG-LTAK63_lo_ induced higher levels of IFN-γ/TNF-α double positive CD4^+^ T cells (Fig. 2d,e). On the other hand, lung cells from mice immunized with rBCG-LTAK63_lo_ displayed higher production of IFN-γ, IL-6, and IL-17 when compared with lung cells from mice immunized with BCG or rBCG-LTAK63_hi_ (Fig. 2f–i).

**Figure 3:**
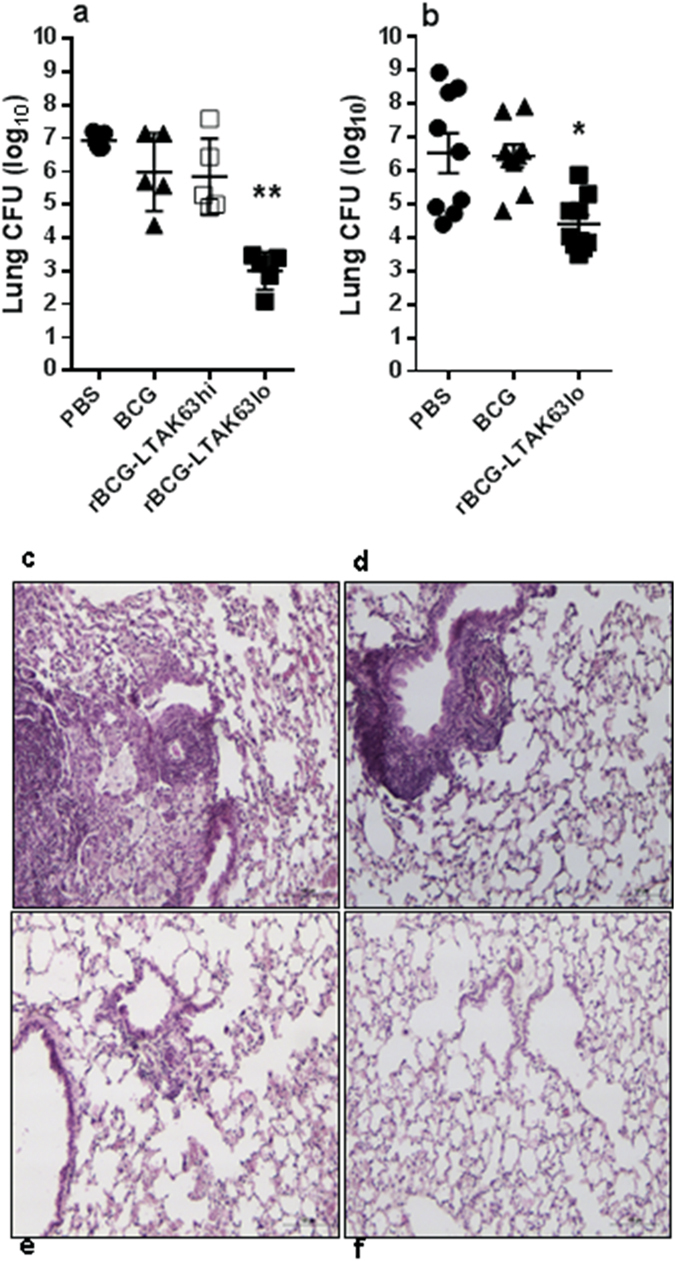
Protection against challenge with Mtb induced by immunization with rBCG-LTAK63_lo_. BALB/c mice were immunized with BCG, rBCG-LTAK63_hi_ or rBCG-LTAK63_lo_ (1 × 10^6^ CFU) and a group received PBS; they were challenged intratracheally 12 weeks later with a dose of Mtb H37Rv (1 × 10^5^ CFU). Bacteria were recovered from the lungs 30 days after challenge. (**a**) Experiment 1 (n = 5 animals) and (**b**) Experiment 2 (n = 8–10 animals). Bars are mean ± S.D. (*) Differences were considered statistically significant when *P* ≤ 0.05 or (**) *P* ≤ 0.01 as compared to the BCG group (one-way Anova). Histological analysis was performed on the lungs of challenged mice from Experiment 1: that received (**c**) PBS, (**d**) BCG, (**e**) rBCG-LTAK63_hi_ or (**f**) rBCG-LTAK63_lo_ and challenged with Mtb 90 days post immunization. Tissues were collected 30 days post challenge, sectioned, stained with H&E, and examined by optical microscopy. Bars indicate 100 µm scale.

BALB/c mice immunized with the rBCG-LTAK63_lo_ construct and challenged with Mtb had a 3.0–4.0 log reduction in CFU (recovered 30 days latter) when compared with control animals and 2.5–3.0 log reduction when compared with BCG-immunized mice (Fig. 3a,b). Histopathology analysis of lung tissues from non-immunized and challenged mice shows intense infiltration of inflammatory cells. The lung tissues of mice immunized with rBCG-LTAK63_lo_ display decreased inflammation as compared to those immunized with BCG (Fig. 3c–f). These findings demonstrate that immunization with rBCG-LTAK63_lo_ induces protective immunity against Mtb and that this may be associated with reduced infection-induced inflammation in the lungs.

### Challenge of rBCG-LTAK63 immunized mice with a higher dose of Mtb

**Figure 4:**
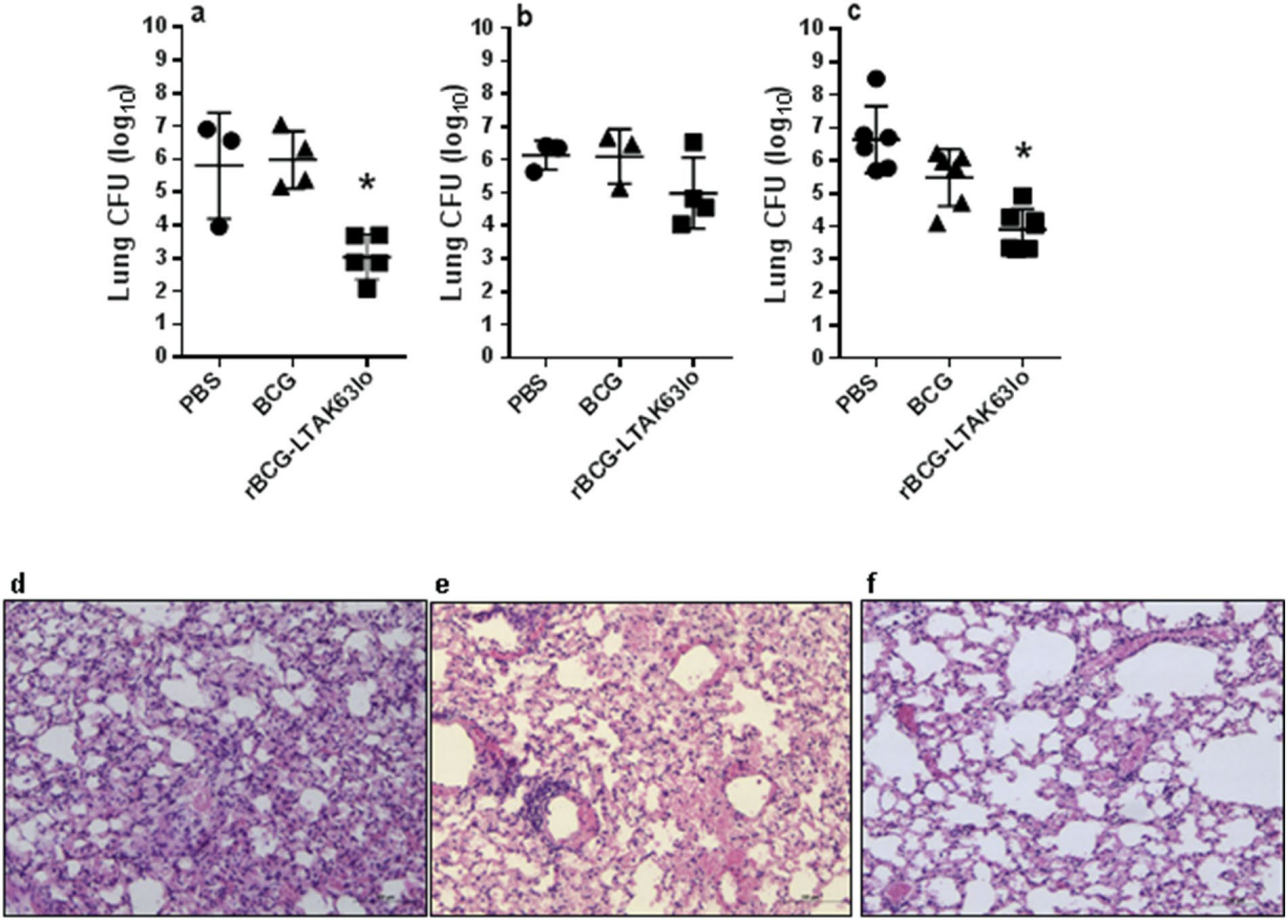
Immunization of mice with rBCG-LTAK63_lo_ induces protection against challenge with a higher dose of Mtb. BALB/c mice were immunized with BCG or rBCG-LTAK63_lo_ (1 × 10^6^ CFU) and a group received PBS and challenged 12 weeks later with a high intratracheal dose of Mtb H37Rv (1 × 10^7^ CFU). Bacteria were recovered from the lungs of challenged mice after (**a**) 30 days or (**b**) 60 days. In a second experiment under the same conditions bacteria were recovered from the lungs of challenged mice after **c**) 120 days. (*) Differences were considered statistically significant when *P* ≤ 0.05 as compared to the BCG group (one-way Anova). Histological analysis was performed on the lungs of challenged mice that received: (**d**) PBS, (**e**) BCG or (**f**) rBCG-LTAK63_lo_. Tissues were collected 30 days post challenge, sectioned, stained with H&E and examined by optical microscopy. Bars indicate 100 µm scale.

BALB/c mice immunized with the rBCG-LTAK63_lo_ construct were challenged after 12 weeks with a 100-fold higher dose of Mtb (1 × 10^7^ CFU of H37Rv) and the bacteria were quantified in the lungs of mice 30 and 60 days after challenge. At 30 days, immunization with BCG provided no protection, while rBCG-LTAK63_lo_ induced a significant reduction of ~3.0 logs CFU in the lungs of immunized mice compared with PBS or BCG-immunized mice (Fig. 4a). All mice immunized with rBCG-LTAK63_lo_ survived, whereas 40% of control or BCG immunized mice died 2 weeks after the Mtb challenge (not shown). At 60 days, mice immunized with rBCG-LTAK63_lo_ still displayed ~1.0 log reduction in CFU in the lungs, while BCG was comparable to control mice (Fig. 4b).

In another experiment, groups of BALB/c mice were immunized with either BCG, rBCG-LTAK63_hi_ or rBCG-LTAK63_lo_, and challenged after 90 days with a high dose of Mtb, and bacteria recovered from the lungs after 120 days. Mice immunized with PBS or BCG had comparable CFU levels, while mice immunized with r-BCG-LTAK63_lo_ showed a 1.1 log reduction when compared with BCG-immunized mice (Fig. 4c).

Histopathology analysis of lung tissues from mice immunized with rBCG-LTAK63_lo_ and challenged with Mtb display decreased inflammation when compared with non-immunized or BCG-immunized mice, which showed intense infiltration of inflammatory cells (Fig. 4d–f).

**Figure 5:**
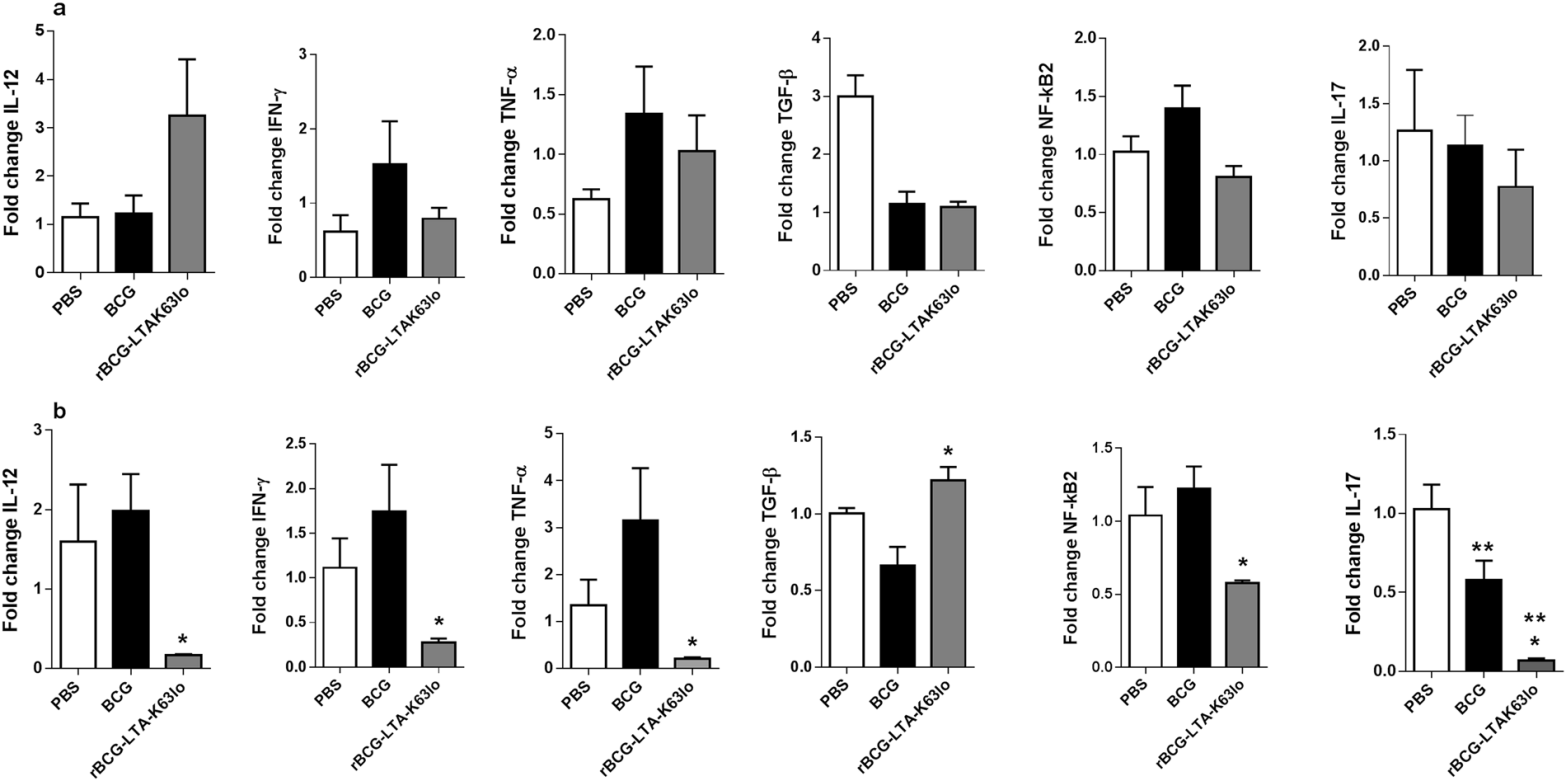
Cytokine production in the lungs of BALB/c mice immunized with rBCG-LTAK63_lo_ and challenged with Mtb as evaluated by Real-Time RT-PCR. Lung tissue from mice immunized with BCG or rBCG-LTAK63_lo_ were collected 30 days post-challenge with either (**a**) Mtb H37Rv (1 × 10^5^ UFC) or (**b**) a higher dose of Mtb H37Rv (1 × 10^7^ UFC) and cytokine mRNA expression fold changes of IL-12, IFN-γ, TNF-α, TGF-β, NF-κB2 and IL-17, were analyzed by Real-Time RT-PCR. Differences were considered statistically significant when *P* ≤ 0.05 as compared to (*) BCG or (**) compared to PBS (one-way Anova). Target gene expression was normalized as compared to GAPDH and Actin levels.

Quantitative mRNA expression of cytokines was determined in the lungs of mice immunized with rBCG-LTAK63_lo_ and challenged with either 1 × 10^5^ CFU (from Fig. 3a) or the higher dose of Mtb, 1 × 10^7^ CFU (from Fig. 4a). When challenged with the higher dose of Mtb, an increased expression of the regulatory molecule, TGF-β, was observed in the rBCG-LTAK63_lo_ group when compared with the BCG group (Fig. 5b), but comparable levels were detected in the low dose challenge (Fig. 5a). On the other hand, expression of the inflammatory cytokines, IL-12, IFN-γ, TNF-α, and IL-17, together with NF-κB2, were decreased in the rBCG-LTAK63_lo_-immunized mice when compared with those immunized with BCG (Fig. 5b), and the same trend was observed for most of the cytokines in the low challenge dose, although not statistically significant (Fig. 5a). In contrast, the expression of TGF-β and the inflammatory cytokines, IL-12, IFN-γ, TNF-α or NF-κB2 were not altered in the BCG group when compared with the PBS group at either challenge dose (Fig. 5a and b). The expression of IL-17 was lower in the BCG group than in the PBS group in the high dose challenge, although IL-17 levels in the rBCG-LTAK63_lo_ were even lower (Fig. 5b).

### Challenge of rBCG-LTAK63 immunized mice with a highly virulent Beijing clinical isolate

**Figure 6:**
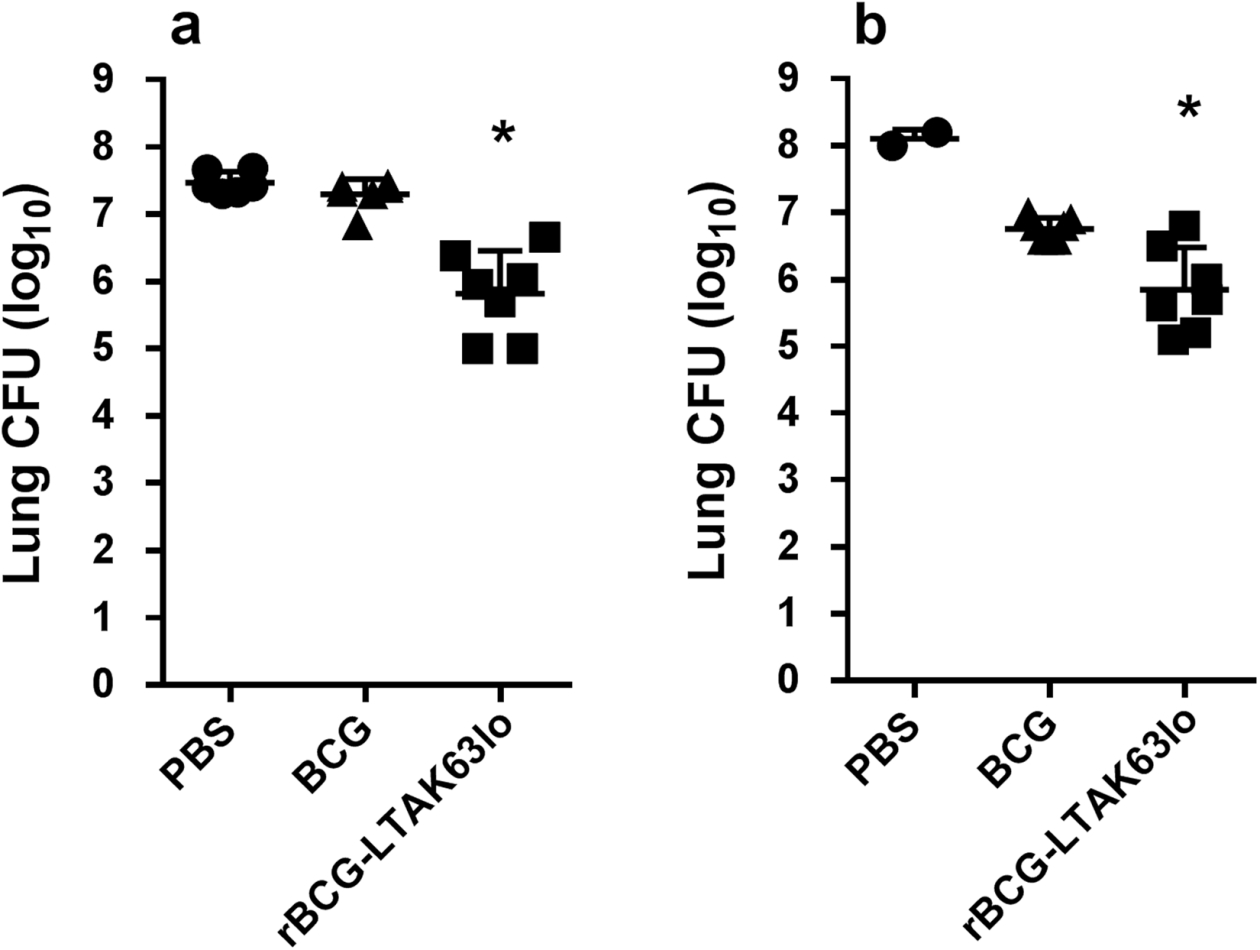
Protection induced by immunization with rBCG-LTAK63_lo_ against challenge with Mtb Beijing clinical isolate. BALB/c mice were immunized with BCG or rBCG-LTAK63_lo_ (1 × 10^6^ CFU) and a group received PBS and they were challenged intratracheally 12 weeks later with a dose of Mtb Beijing clinical isolate (1 × 10^3^ CFU). Bacteria were recovered from the lungs 30 days after challenge. (**a**) Experiment 1 (n = 7 animals) and (**b**) Experiment 2 (n = 7 animals). Bars represent mean ± S.D. (*) Differences were considered statistically significant when *P* ≤ 0.05 as compared to the BCG group (one-way Anova).

In order to assess the efficacy of rBCG-LTAK63_lo_ vaccine against a clinical isolate, BALB/c mice immunized with rBCG-LTAK63_lo_ or BCG, were challenged 90 days later with the Mtb Beijing strain 1471. The dose chosen (1 × 10^3^ CFU/animal) was previously determined as a lethal dose in C57BL/6 mice^27^. An examination of lung bacterial load 30 days later showed that mice immunized with rBCG-LTAK63_lo_ had 1.5–2.2 logs reduction in bacterial burden when compared to mice immunized with BCG or PBS (*P* < 0.05) (Fig. 6).

## Discussion

The significant new findings of this study are the demonstration that a modified BCG vaccine expressing a non-toxic mutant toxin of *E*. *coli*, with adjuvant properties, is highly immunogenic and can confer superior protection against *M*. *tuberculosis* and a subsequent regulatory mechanism after infection that prevents pathology in the lungs. We have previously demonstrated that BCG expressing bacterial toxin derivatives with known adjuvant properties can shift immune responses induced towards a Th1-type *in vivo* ^19, 20^. Although there is still considerable controversy over what kind of immune response is required for an effective vaccine against tuberculosis, the consensus view is that a shift towards a Th1 profile should be protective, with increased production of IFN-γ, TNF-α and IL-2. More recently there is evidence of a protective role for IL-17 and multifunctional CD4^+^ T cells^4, 28^. In this study, we demonstrate significant protection against TB challenge in mice using a recombinant BCG expressing the mutated A subunit of LTK63, exploiting the known adjuvant properties of LT.

The rBCG-LTK63 was not stable, but using only the LTAK63 subunit expressed under the control of 2 different promoters (a strong and a weak mycobacterial promoter) it was possible to obtain different expression levels – rBCG-LTAK63_hi_ and rBCG-LTAK63_lo_. The level of antigen expression in BCG can affect the immune response induced^29^ and this could influence the intensity of the adjuvant effect on the mycobacteria and possibly the level of protection. The expression of the A subunit through a high-strength promoter allowed stable expression, but induced lower protection levels against challenge. Higher protection levels were obtained through the use of a weaker mycobacterial promoter. Analysis of the antigen-specific immune response using spleen cells from immunized mice revealed IFN-γ and TNF-α production, even at 60 days after immunization, but this did not correlate with the protection. In contrast, evaluation of immune responses in the lungs revealed higher IFN-γ, TNF-α, IL-6 and IL-17 production in rBCG-LTAK63_lo_- immunized mice when compared with BCG or rBCG-LTAK63_hi_ groups and this correlated with better protection against challenge with *M*. *tuberculosis*. However, more complex mechanisms may be involved and other parameters should be investigated to serve as correlates of protection^30^.

Immunization of mice with either construct of rBCG-LTAK63 induced higher Th1 responses when compared to the BCG group, determined either by the higher concentrations of IFN-γ and/or TNF-α produced by splenocytes from immunized animals or by IFN-γ/IL-2 or IFN-γ/TNF-α double positive CD4^+^ T cells. The immune responses detectable in the spleen were comparable for both rBCG-LTAK63 constructs, and were not very different from those reported for other vaccine candidates based on recombinant BCG, or even other vaccine strategies^31^. It was only in the lungs that clear differences in the immune response could be observed between the different BCG vaccines, probably reflecting the importance of local cellular immune responses at the site of infection.

The two most widely used Mtb challenge models have been the aerosol and the intratracheal models, and authors have favored the first due to the fact that it would be a more physiological representation of infection. However, it has been hypothesized that, the low dose challenge may not represent the high and constant exposure of individuals to Mtb infection occurring in high burden settings^32^. This could account for the inability of the currently used challenge models to provide correlates of protection and predict efficacy in humans. Nonetheless, whichever model used, at 30 days after infection, mice usually have comparable numbers of bacteria in the lungs ~10^6^–10^7^ CFUs, showing a 1 log reduction due to BCG immunization. The current strategies being investigated for the development of vaccines against TB have shown variable results in terms of protection. Strategies based on live attenuated bacteria or viruses, expressing immunodominant antigens from Mtb, or BCG prime/Mtb protein boost have been shown to induce up to 1 log CFU reduction as compared to BCG^7^. The immunization of mice with rBCG-LTAK63_lo_ showed a protection level considerably higher than those described to date, inducing a 2.0–3.0 log reduction in CFU in the lungs of immunized and challenged mice. Furthermore, histopathological analysis indicated considerable reduction in lung injury as compared to BCG.

On the other hand, in these models BCG is protective in adult mice, which does not reflect what is observed in adult humans, usually not protected by immunization with the BCG vaccine. Rook *et al*.^32^ have proposed that populations living in areas with higher burden of disease such as developing countries, may be exposed to different conditions of infection, which may not be reproduced by our animal challenge models. They hypothesize that probably due to overcrowding and delayed treatment much of TB occurring is due to high-dose challenge, and thus higher challenge doses should be investigated^32^. Therefore, we have used a more stringent model, inoculating higher doses of the Mtb H37Rv strain in a challenge condition in which BCG is not protective. Under these challenge conditions, wild type BCG showed no protection in terms of lung colonization by Mtb when compared with control animals. Since rBCG-LTAK63_lo_ was more immunogenic and protective, it was used for further characterization. Animals immunized with the rBCG-LTAK63_lo_ construct, displayed 100% survival following the high-dose challenge with 10^7^ CFU up to 120 days after immunization, while both BCG and non-immunized animals had a fatality rate of 40%, probably due to non-controlled inflammation as observed by histopathology analysis (not shown). The fact that mice immunized with rBCG-LTAK63_lo_ show a strong immune response in the lungs at 60 days and significant protection at 90 days after immunization indicates induction of a long-term memory immune response. Induction of memory immune responses will be further investigated.

We investigated the immune response induced in the lungs of immunized animals after intratracheal challenge with 2 different challenge doses of Mtb, 10^5^ CFU and 10^7^ CFU. Mice that were immunized with rBCG-LTAK63_lo_ and challenged with the higher Mtb dose were shown to have a suppressive lung environment, with decreased levels of Th1 and IL-17 cytokines, as detected by real time PCR in the lungs, and a similar tendency was observed at the lower dose challenge. This effect seems to be regulated by TGF-β. This is contrary to what was observed in BCG-immunized animals. This cytokine has been shown to be up-regulated in inflammatory processes against bacterial infections^33^. Furthermore, TGF-β has also been implicated in the inhibition of mycobacterial growth^34^. Our results show that immunization with rBCG-LTAK63_lo_ induces a more intense Th1 response profile and IL-17 cytokine production in the lungs and suggests a mechanism of protection associated with limitation of the inflammatory response after challenge that prevents immunopathology. Whether it was the higher immune response or other mechanisms induced by the vaccine itself that triggered the regulatory response after infection is still to be determined. This regulatory response induced after challenge, with reduction in Th1 and inflammatory cytokines, is an effect that has not been observed with BCG or with other Mtb vaccine candidates.

Clinical Mtb isolates are useful to verify whether vaccine candidates can be effective towards MDR strains. Here we showed that rBCG-LTAK63_lo_ was protective against this hypervirulent Beijing strain, whereas BCG did not have any protective effect. Interestingly, it has been shown that BCG offers protection against some Beijing strains, but not others, and the mechanisms are yet to be determined^35^. The variability of virulence among Beijing strains could be crucial for effective host protection against Mtb infection mediated by BCG^36, 37^. Furthermore, immunization with rBCG-LTAK63_lo_ induced a 1.0–2.0 log reduction in CFU of mice challenged with the Beijing strain as compared to BCG, which is lower than the 2.0–3.0 log reduction observed when mice are challenged with H37Rv, indicating that protection would be higher against the latter strain. It has been described that the mechanism of infection of H37Rv and Beijing strains can be different^27^.

There are several strategies currently being pursued for the development of TB vaccines based on live mycobacterial strains (either recombinant BCG or attenuated *M*. *tuberculosis*)^9, 38, 39^. Strategies based on BCG improvement by overexpression of MTB immunodominant antigens, such as Ag85B or the RD-1 locus, have been seriously considered for further development^38, 40^. Phagosome-escape mutants incorporating Listeriolysin of *Listeria monocytogenes* have been shown to be more effective in eliciting an immune response against TB, progressing to Phase II clinical trials with promising results^39, 41^. Recently, it has been shown that the deletion of *zmp1* gene from BCG improved protection in the guinea pig model of tuberculosis^42^. However, the lack of immune correlates of protection for MTB in validated animal models or in humans has hindered progress. Furthermore, the use of different immunization regimes and infection models has limited the comparison of protective efficacy between the different vaccine candidates. Nevertheless, considering the reduction in bacterial load recovered in the lungs after challenge, it is clear that rBCG-LTAK63_lo_ displays extremely high protection levels; some parameters support our conclusion: (1) the comparison with wild type BCG, (2) the histopathological analyses of the lungs after the challenge, which show preserved tissue even after a very high challenge dose and (3) although the intratracheal challenge doses used are much higher than those used in aerosol challenges, the bacterial burden recovered from non-immunized and BCG control groups are comparable. This strain can be the basis for expression of other immunogenic TB antigens, in an attempt to further increase its protective properties. On the other hand, due to the high protection levels, it would be suitable for Systems Biology studies in search for much needed biomarkers of protection against TB^43^.

The Geneva consensus in 2005 congregated the current experiences with live mycobacterial vaccines to identify essential steps in the development of new live TB vaccines^44^. A set of quality and safety requirements were established to guide product development^44^. The second Geneva Consensus in 2009 outlined regulatory requirements, manufacturing considerations and general criteria for clinical development towards Phase I, II and III trials^45^. Both consider that it is essential to provide a complete characterization of the product and establish consistent protective efficacy in animal models. Here, we initiate the process, characterizing the immune response and protective efficacy of the rBCG-LTAK63_lo_ strain. Experiments to characterize the safety of the strain and the protective efficacy in other animal models in the aerosol challenge are underway.

In the current study, we have demonstrated the importance of promoter strength and level of expression of the LTK63 adjuvant derivatives expressed in recombinant BCG on the modulation of the immune response induced against mycobacteria. The superior immunogenicity and protection induced by this recombinant BCG strain against Mtb challenge revealed a novel mechanism of protection against pathology after infection.

## Methods

### Bacterial strains and Mtb challenge

The *Mycobacterium bovis* BCG Moreau strain (Instituto Butantan) was used to generate the recombinant BCG strains; *M*. *tuberculosis* H37Rv and Beijing clinical isolate 1471 were used in the challenge experiments (Supplementary Methods).

All animal experiments were performed according to Brazilian and international guidelines on animal experimentation and approved by the Ethics Committee at Instituto Butantan, São Paulo – SP (CEUAIB), (Permit Number 601/09). Mice were challenged by the intratracheal route with 1 × 10^5^ CFU of Mtb per animal, as a dose previously established in the laboratory^46^. Alternatively mice were challenged with a higher dose, 1 × 10^7^ CFU of Mtb per animal, 60 or 90 days after a single immunization. Immunized mice were also challenged with 1000 CFU Beijing isolate, as previously described^27^. Animals were euthanized 30, 60 or 120 days after the infection and the bacterial loads were determined by plating whole or partial lung homogenates on MB7H10/OADC agar plates.

### Flow cytometry for cell-surface markers and intracellular cytokines

Lung cells and/or splenocytes (2 × 10^5^) were isolated (Supplementary Methods) stimulated with CFP (5.0 µg/mL) for 12 h at 37 °C and 5% CO_2_. The cells were then collected for intracellular cytokine staining with FITC, PE or PE-Cy7-conjugated monoclonal antibodies against CD4 and cytokines. The supernatant was collected for analysis by Cytometric Bead Array (BD Biosciences, San Diego, CA) Mouse Th1/Th2/Th17 Cytokine Kit or by Enzyme-linked immunosorbent assay (ELISA). Data were acquired on a FACSCanto II flow cytometer (BD) and analyzed using the FlowJo 8.7 software.

### Real-time reverse transcription-polymerase chain reaction (qPCR)

Lung cell suspensions were recovered from immunized animals 30 days after challenge with Mtb. Total RNA was isolated using a Nucleospin II kit, according to the manufacturer’s directions (BD Biosciences). The mRNA was reverse transcribed using a ThermoScript™ RT-PCR System (Invitrogen, Carlsbad, CA) for First-Strand cDNA Synthesis. Pre-designed gene expression and TaqMan Gene Expression Master Mix (Invitrogen) were used with the Applied Biosystems (Foster City, CA), 7300 Real-Time PCR apparatus. Target gene expression was normalized to GAPDH and actin levels.

### Statistical analysis

Results were expressed as mean (±) SD of at least two independent experiments. Significance of differences among groups was calculated by Student´s *t* tests or ANOVA.

## Electronic supplementary material

Supplementary Information
