# Supplementary material for: Recombinant BCG Expressing LTAK63 Adjuvant induces Superior Protection against Mycobacterium tuberculosis
Source: Sci Rep. 2017 May 18;7:2109. doi: 10.1038/s41598-017-02003-9 (PMC5437048; doi:10.1038/s41598-017-02003-9)
Supplement: Supplementary file 1 — Supplementary Information [file 41598_2017_2003_MOESM1_ESM.pdf]

Recombinant BCG Expressing LTAK63 Adjuvant induces Superior Protection against *Mycobacterium tuberculosis*

Ivan P. Nascimento[1], Dunia Rodriguez[1], Carina C. Santos[1,4], Eduardo P. Amaral[2], Henrique K. Rofatto[3], Ana P. Junqueira-Kipnis[5], Eduardo D.C. Gonçalves[6], , Maria R. D'Império-Lima[2], Mario H. Hirata[7], Celio L. Silva[8], Nathalie Winter[9], Brigitte Gicquel[10], Kingston H.G. Mills[11], Mariagrazia Pizza[12], Rino Rappuoli[12], Luciana C.C. Leite[1*]

Supplementary Information

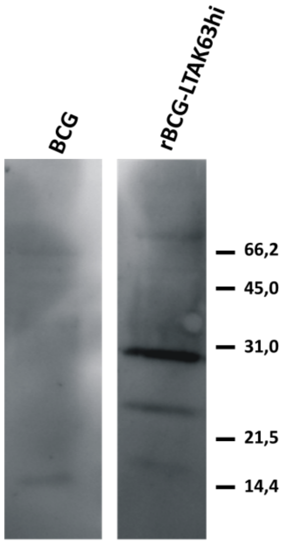

Supplementary Figure 1. **Expression of LTAK63 in BCG by Western blot.** Total soluble protein from BCG or rBCG-LTAK63$_{hi}$ (30 µg) were analyzed by Western blot using an anti-LT polyclonal antibody. The samples were processed at the same time and images were derived from the same SDS-PAGE and Western blot.

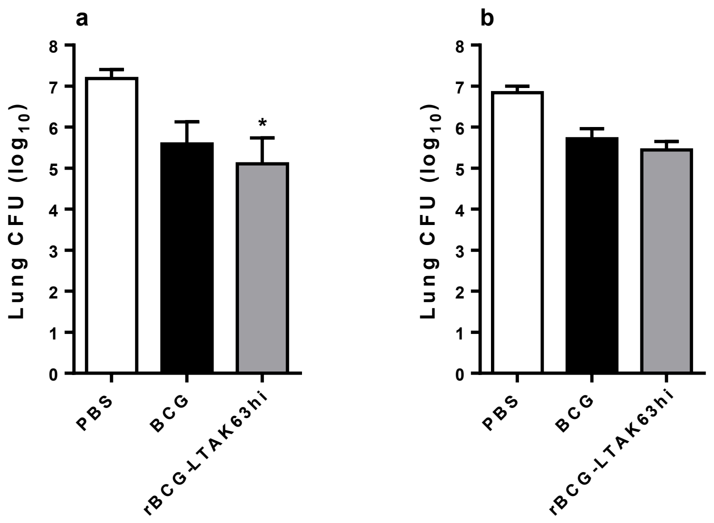

Supplementary Figure S2. Protection against challenge with Mtb induced by immunization with rBCG-LTAK63$_{hi}$. Mice were immunized with rBCG-LTAK63$_{hi}$ or BCG (1×10^6 CFU) and challenged 8 weeks later intratracheally with a dose of Mtb H37Rv (1×10^5 CFU). Bacteria were recovered from the lungs 30 days after challenge. **a**) Experiment 1 (n=10 animals) and **b**) Experiment 2 (n=10 animals). Significant differences were determined by Student´s t-test. Results are mean ± S.D. (*) Differences were considered statistically significant when $P \leq 0.05$ as compared to the BCG group.

Supplementary Table S1. Plasmids and promoters used in this study

Plasmids/Promoters	Description	Reference
pMIP12	Mycobacterial shuttle vector containing Mega Shine Dalgarno, P_{BlaF*} promoter, Kanamycin resistance, 6,000 pb	Ref. 2, 3
pLA71	Mycobacterial shuttle vector containing P_{BlaF*} promoter, Kanamycin resistance, 12,000 bp	Ref. 3
pMIP12-*ltk63*	pMIP12, carrying *lta-k63*-SD-*ltb*	This study
pLNIP-*ltka63*	pMIP12, carrying *lta-k63*	This study
pAN71-*ltak63*	pLA71, substituting P_{AN} promoter for P_{BlaF*}, carrying the codon optimized *lta-k63*	This study
P_{AN}	*M. paratuberculosis* promoter	Ref. 4

Supplementary Table S2. Primers used to construct the expression vectors

Gene/Sequence	Primers	Restriction sites [a]
ltk63 [b]	Forward: 5'–TAGGGTACCCAAAAATATAACTTCATTTTTTTATTTT–3′	*Kpn*I
	Reverse: 5'–TAGCTGCAGCTAGTTTTTCATACTGATTGCCG–3'	*Pst*I
ltak63 [c]	Forward: 5′–TAGGGATCCAATGGCGACAGATTATACCGTTG–3′	*BamHI*
	Reverse: 5′–TAGGGTACCTAATTCATCCCGAATTCTGTTATA–3′	*KpnI*
P_{AN} [d]	Forward: 5′–TAGGGATCCGATCCCGTGACAAGGCC–3'	*BamH*I
	Reverse: : 5′–TAGGGTACCCATTGAGAATCTCCTTCTGGG–3′	*Kpn*I

[a] Restriction sites are underlined; [b] primers used for cloning the *ltk63* in pMIP12; [c] primers used for cloning the *ltak63* in pMIP12, [d] primers used for cloning the P_{AN} promoter in pLA71.

Supplementary Table S3. Sequence of the optimized *ltak63* gene codon [a]

[b]GGTACCAACGGCGACCGCCTCTATCGGGCCGACTCGCGTCCGCCGGATGAGATCAAACGGT
CCGGCGGTCTGATGCCGCGTGGCCACAACGAGTACTTCGACCGCGGTACGCAGATGAACAT
CAATCTGTACGACCACGCCCGTGGGACGCAGACCGGCTTCGTCCGCTATGACGACGGTTACG
TGTCGACCAAGCTGAGCCTGCGCTCGGCCCATCTGGCCGGACAATCCATCCTGTCCGGCTAC
TCCACGTACTACATCTACGTGATTGCGACCGCCCCAAACATGTTCAATGTGAACGACGTCCT
GGGCGTCTACAGCCCCCATCCCTATGAGCAGGAAGTGAGCGCCCTCGGCGGGATTCCCTATA
GCCAGATCTACGGTTGGTATCGGGTCAACTTTGGAGTGATCGACGAGCGCTTGCACCGGAAC
CGTGAATATCGCGATCGGTACTACCGCAACCTGAACATCGCGCCAGCGGAAGATGGCTACC
GCTTGGCCGGGTTCCCACCGGACCACCAAGCGTGGCGGGAGGAGCCGTGGATTCACCACGC
GCCACAGGGCTGTGGCAATTCCTCGCGGACCATTACCGGGGACACCTGCAACGAGGAAACG
CAGAACCTCAGCACGATCTATTTGCGGGAGTACCAGTCGAAGGTGAAGCGCCAAATCTTCTC
GGACTACCAGTCGGAGGTCGACATCTACAACCGCATCCGGGATGAACTGTGAGCGGCCGC[c]

This sequence was commercially synthetized and the restriction sites *KpnI*[b] and *NotI*[c] added for cloning directly into the pAN71 vector.

Supplementary Methods

Preparation of expression vectors and recombinant BCG strains. The genetically detoxified *ltak63* and the *ltb* genes were amplified from a template p-Bluescript vector containing the genes as previously described[1]. The mycobacterial expression vectors pMIP12 and the modified pLA71 were previously described (Supplementary Table 1) [2,3]. These vectors originally contained the *E. coli* and mycobacterium origins of replication, a kanamycin resistance gene, an ATG initiation codon followed by a multiple cloning site, and the up-regulated *M. fortuitum* promoter P_{blaF*}, containing the signal sequence of ß-lactamase, in the case of pLA71; or the optimized Shine-Dalgarno (SD) sequence in the case of pMIP12. Here, we constructed a new expression vector called pAN71, by modification of the pLA71 vector, replacing the original expression cassette for the P_{AN} promoter, without the signal sequence of ß-lactamase (Supplementary Tables 1 and 2)[4].

The whole *ltk63* gene was cloned into the pMIP12 vector, including a SD sequence between *ltak63* and *ltb*, resulting in the pMIP-*ltk63* plasmid (Supplementary Tables 1 and 2). Transformation of BCG with this vector generated rBCG-LTK63. A second construct placed only the *ltak63* gene under the control of P_{blaF*} in fusion with the ß-lactamase signal sequence, generating pLNIP-*ltak63* (Supplementary Tables 1 and 2) and BCG transformed with this vector produced rBCG-LTAK63$_{hi}$. Another construct placed a codon optimized *ltak63* gene (Supplementary Table 3) under control of the P_{AN} promoter in the modified expression cassette pAN71, generating pAN-*ltak63* (Supplementary Tables 1 and 2), which upon transformation of BCG produced rBCG-LTAK63$_{lo}$.

SDS-PAGE and Western blotting

One or more rBCG cultures clones were harvested and disrupted with an Ultrasonic Processor GE 100. Aliquots of protein extracts (~10 µg) were separated by SDS-PAGE and electrotransferred onto a PVDF membrane. The presence of LTK63 or LTAK63 was detected using a rabbit polyclonal antiserum (1:1,000) raised against LT (kindly provided by Dr. Roxane Piazza, Instituto Butantan, São Paulo, Brazil) or an ascite mouse or monoclonal anti-LTAK63 antibody. The immunoblots were developed with goat anti-mouse or anti-rabbit peroxidase-conjugated antibodies (1:1,000) and visualized with an ECL-Plus kit (GE Healthcare, Sweden).

Lung and spleen cell preparation and cytokine assays

Sixty or ninety days after immunization, mice were euthanized and their lungs and/or spleens isolated (Supplementary Methods). Cytokine production was evaluated by ELISA or CBA, following manufacturer's instructions (Peprotech, Rocky Hill, NJ) and/or (BD Biosciences). Briefly, cells (2×10^5/well) from the spleen and/or lungs of individual animals in 96-well plates were incubated with CFP (5.0 μg/mL) for 48 h at 37 ^oC. CFP is a concentrate (5,000 kDa cut off) from culture filtrates of mycobacterial protein derived from the supernatant of *M. bovis* BCG grown on Sauton medium for 14 days.

References

1 Pizza, M. *et al.* Mucosal vaccines: non toxic derivatives of LT and CT as mucosal adjuvants. *Vaccine* **19**, 2534-2541, doi:Doi 10.1016/S0264-410x(00)00553-3 (2001).

2 Le Dantec, C., Winter, N., Gicquel, B., Vincent, V. & Picardeau, M. Genomic sequence and transcriptional analysis of a 23-kilobase mycobacterial linear plasmid: evidence for horizontal transfer and identification of plasmid maintenance systems. *Journal of bacteriology* **183**, 2157-2164, doi:10.1128/JB.183.7.2157-2164.2001 (2001).

3 Lim, E. M. *et al.* Identification of Mycobacterium-Tuberculosis DNA-Sequences Encoding Exported Proteins by Using Phoa Gene Fusions. *Journal of bacteriology* **177**, 59-65 (1995).

4 Murray, A. *et al.* Expression of Escherichia-Coli Beta-Galactosidase in Mycobacterium-Bovis Bcg Using an Expression System Isolated from Mycobacterium-Paratuberculosis Which Induced Humoral and Cellular Immune-Responses. *Mol Microbiol* **6**, 3331-3342, doi:DOI 10.1111/j.1365-2958.1992.tb02201.x (1992).
